# Narrow-band UVB radiation promotes dendrite formation by activating Rac1 in B16 melanoma cells

**DOI:** 10.3892/mco.2013.145

**Published:** 2013-07-11

**Authors:** WU-QING WANG, JIN-FENG WU, XIAO-QING XIAO, QIN XIAO, JING WANG, FU-GUO ZUO

**Affiliations:** 1 Department of Dermatology, The Central Hospital of Minhang, Shanghai 201199, P.R. China; 2 Department of Dermatology, Huashan Hospital, Fudan University, Shanghai 200040, P.R. China; 3 Department of Dermatology, East Hospital, School of Medicine, Tongji University, Shanghai 200120, P.R. China

**Keywords:** narrow-band ultraviolet B radiation, Rac1, F-actin rearrangement, dendricity, B16 melanoma cells

## Abstract

Melanocytes are found scattered throughout the basal layer of the epidermis. Following hormone or ultraviolet (UV) light stimulation, the melanin pigments contained in melanocytes are transferred through the dendrites to the surrounding keratinocytes to protect against UV light damage or carcinogenesis. This has been considered as a morphological indicator of melanocytes and melanoma cells. Small GTPases of the Rho family have been implicated in the regulation of actin reorganization underlying dendrite formation in melanocytes and melanoma cells. It has been proven that ultraviolet light plays a pivotal role in melanocyte dendrite formation; however, the molecular mechanism underlying this process has not been fully elucidated. The effect of small GTPases, such as Rac1 and RhoA, on the morphology of B16 melanoma cells treated with narrow-band UVB radiation was investigated. The morphological changes were observed under a phase contrast microscope and the F-actin microfilament of the cytoskeleton was observed under a laser scanning confocal microscope. The pull-down assay was performed to detect the activity of the small GTPases Rac1 and RhoA. The morphological changes were evident, with globular cell bodies and increased numbers of tree branch-like dendrites. The cytoskeletal F-actin appeared disassembled following narrow-band UVB irradiation of B16 melanoma cells. Treatment of B16 melanoma cells with narrow-band UVB radiation resulted in the activation of Rac1 in a time-dependent manner. In conclusion, the present study may provide a novel method through which narrow-band UVB radiation may be used to promote dendrite formation by activating the Rac1 signaling pathway, resulting in F-actin rearrangement in B16 melanoma cells.

## Introduction

Melanocytes are derived from the neural crest and are found scattered throughout the basal layer of the epidermis. In response to hormones or ultraviolet (UV) light, the melanin pigments contained in melanocytes are transferred to the surrounding keratinocytes through the tips of the dendrites to protect against UV light damage or carcinogenesis. This has been considered a morphological indicator of melanocytes and melanoma cells. During this process, dendrite extension is critical, since melanocytes constitute a minor population in the epidermis (1–3). Previous studies demonstrated that the dendritic outgrowth of melanocytes and melanoma cells is promoted by UV light irradiation, growth factors and cAMP-elevating agents (4–6).

The rearrangement of the actin cytoskeleton promoting dendritic outgrowth and the underlying signaling mechanisms in melanocytes and melanoma cells were previously investigated (3,4,7). The identification of the small GTPases of the Rho family has elucidated the molecular signaling underlying cell shape alterations. The small GTPases of the Rho family regulate several intracellular processes, including cytoskeletal reorganization, cell motility, cell-cell adhesion and apoptosis (8–11), as well as regulation of the cell cycle, oncogenesis and gene transcription (12,13). Twenty genes encoding proteins belonging to the Rho family of small GTPases have been described in humans thus far. With regards to cell morphology, three of the small GTPases appear to be more important: Rac1, RhoA and Cdc42 (14). The role of Rac1 and RhoA in dendrite formation by human melanocytes and melanoma cells was defined by several studies. Overexpression of the constitutive active form of MKK6 resulted in significant elongation of the dendrites via the upregulation of Cdc42 and Rac1 expression in the SK-mel-24 human melanoma cell line (15). A study conducted by Ito *et al*(16) demonstrated that centaureidin may act via the Rho signaling pathway to inhibit dendrite outgrowth in melanocytes. Furthermore, Scott and Leopardi (17) reported that cAMP-mediated dendrite formation in B16 melanoma cells occurs via the upregulation of Rac and inhibition of Rho activity.

There is substantial evidence suggesting that UV irradiation plays a pivotal role in regulating melanocyte dendricity. In the development of melasma, the strongest predictors are UV light exposure and genetic factors (18,19). Furthermore, the histological analysis of melasma lesions revealed that epidermal melanocytes exhibited a strong staining intensity and more dendrites (20). Reflectance confocal imaging of murine skin *in vivo* following exposure to UVB light highlighted the dendricity of melanocytes (3). Although it is likely that UV light is a primary stimulus for melanocyte dendrite formation, the possible molecular mechanisms have not been fully elucidated. The effect of narrow-band UVB radiation on the morphological changes and the actin cytoskeleton in B16 melanoma cells was investigated, with the aim of elucidating the mechanism underlying this process.

## Materials and methods

### Cell culture and narrow-band UVB radiation treatment

B16 melanoma cells are transformed melanocytes and suitable as a physiological model of normal melanocytes. In the present study, B16 melanoma cells were investigated to elucidate the mechanism of dendrite formation induced by narrow-band UVB radiation. B16 melanoma cells (Institute of Biochemistry and Cell Biology, Shanghai Institutes for Biological Sciences, Chinese Academy of Sciences, Shanghai, China) were maintained in RPMI-1640 medium (Gibco, Carlsbad, CA, USA) supplemented with 10% fetal bovine serum (FBS) and antibiotics (100 IU/ml penicillin and 50 mg/ml streptomycin) (Invitrogen, Grand Island, NY, USA) in a humidified atmosphere of 5% CO_2_ at 37ºC. The cells were exposed to various doses of radiation by a specific UVB lamp emitting a peak wavelength of 311 nm with an intensity of 1 mW/cm^2^ (Philips, Amsterdam, Netherlands). During irradiation, the medium was replaced with phosphate-buffered saline (PBS) to avoid the formation of medium-derived toxic photoproducts induced by UVB exposure. Immediately after phototreatment, PBS was removed and media were added to the cells. The subsequent experiments were performed three times in triplicate.

### Cell viability assay

The cells were seeded in 96-well plates at a density of 1×10^4^ cells/well and incubated for 24 h. Subsequently, the cells were exposed to various doses of narrow-band UVB radiation (0, 25, 50, 100, 200 and 300 mJ/cm^2^) and were incubated for an additional 24 h. Cell viability was assessed using a commercially available kit (Cell Counting kit-8; Dojindo Co., Ltd., Kumamoto, Japan) according to the manufacturer’s instructions to determine the appropriate dose of narrow-band UVB radiation for the subsequent experiments. The colorimetric absorbance was recorded at 490 nm using the ELx800 microplate reader (BioTek, Winooski, VT, USA).

### Dendrite formation assay

B16 melanoma cells (1×10^5^ cells/well) growing in 6-well microplates were cultured for 24 h and were subsequently irradiated with narrow-band UVB light (100 mJ/cm^2^). Following incubation for a further 24 h in an FBS-free medium (in order to avoid the effect of growth factors in FBS on dendrite formation), the morphological changes were observed under an ELWD 0.3 phase contrast microscope (Nikon, Tokyo, Japan). The number of dendrites per cell was determined for ~100 cells from each experiment.

### Immunofluorescence microscopy

B16 melanoma cells growing on coverslips were placed within culture plates (1×10^5^ cells), cultured for 24 h and then irradiated with narrow-band UVB light (100 mJ/cm^2^). Following incubation for an additional 24 h in FBS-free medium for immunofluorescence studies, the B16 melanoma cells growing on glass coverslips were briefly rinsed with PBS and fixed in 4% formaldehyde, then permeabilized with 0.5% Triton X-100 (Sigma-Aldrich, St. Louis, MO, USA) for 2 min for detection of cytoskeletal F-actin. The cells were washed for 10 min in PBS and exposed to rhodamine phalloidin staining (Cytoskeleton Inc., Denver, CO, USA) diluted in PBS containing 1% of BSA, applied for 30 min at 37ºC. The coverslips were rinsed with PBS for 10 min and observed and photographed with a Leica TCS SP2 laser scanning confocal microscope (LSCM) (Leica, Solms, Germany).

### Pull-down assay

An equal number of B16 melanoma cells (~1×10^6^ cells) was cultured, followed by lysis in ice-cold cell lysis buffer supplemented with 1X protease inhibitor cocktail after narrow-band UVB irradiation for the indicated time. The pull-down assay was implemented for the detection of GTP-Rac1 and -RhoA using a commercially available kit (Cytoskeleton Inc.) according to the manufacturer’s protocol. In brief, a pre-determined amount of PAK-PBD beads (20 μg) or GST-Rhotekin-RBD beads (10 μg) was added to the cleared lysates and incubated at 4ºC on a rotator for 1 h. GTP-bound proteins were captured onto the beads and pelleted. The beads were rinsed with a wash buffer and GTP-bound protein was eluted with 2X Laemmli sample buffer. Samples were immunoblotted after 12.5% SDS-PAGE using standard procedures. To assess loading equality among different lysates, a portion of each lysate was removed prior to the addition of PAK-PBD beads or GST-Rhotekin-RBD beads and blotted with anti-RhoA and anti-Rac1 antibodies. The expression of GTP-Rac1 and GTP-RhoA was detected using the Plus-ECL method. Densitrometric analysis was performed using LAS-3000 ECL image analysis system (Fujifilm Medical Systems, Tokyo, Japan).

## Results

### Effects of narrow-band UVB radiation on B16 melanoma cell viability

B16 melanoma cells were irradiated with different doses of narrow-band UVB light. The results demonstrated that irradiation with narrow-band UVB light at 25, 50 and 100 mJ/cm^2^ exerted no stimulatory effect on cell proliferation compared to control groups. However, 200 and 300 mJ/cm^2^ of irradiation significantly reduced the cell survival rate. According to these results, narrow-band UVB irradiation at 100 mJ/cm^2^ was selected as the appropriate dose for the subsequent experiments.

### Morphological changes in B16 melanoma cells following narrow-band UVB irradiation

**Figure 1:**
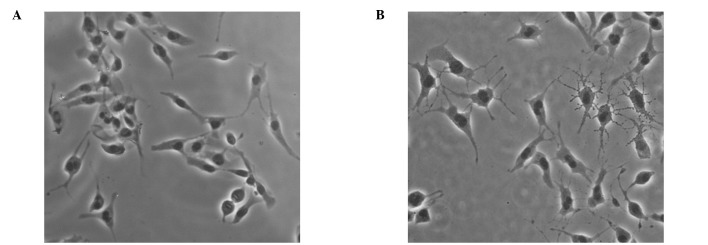
Morphological changes in B16 melanoma cells following narrow-band UVB irradiation. The morphological changes were observed under a phase contrast microscope (bar, 50 μM). (A) Non-irradiated B16 cells exhibit 2–3 dendrites. (B) B16 melanoma cells with globular cell bodies and an increased number of tree branch-like dendrites at 24 h following narrow-band UVB irradiation (100 mJ/cm^2^).

To investigate the effects of narrow-band UVB radiation on dendrite formation, B16 melanoma cells were irradiated with 100 mJ/cm^2^ narrow-band UVB light for 24 h. The dendrite number in 100 B16 melanoma cells from each experiment was manually counted. The morphological changes in B16 melanoma cells induced by narrow-band UVB radiation were evident, with markedly globular cell bodies and significantly increased numbers of tree branch-like dendrites (5.52±1.35/per cell) compared to untreated cells, which exhibited 2–3 dendrites (2.39±0.36/per cell) (Fig. 1).

### Effects of narrow-band UVB radiation on cytoskeletal F-actin in B16 melanoma cells

**Figure 2:**
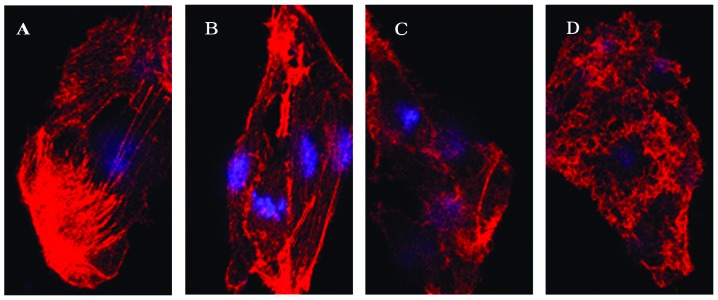
Effects of narrow-band UVB radiation on cytoskeletal F-actin in B16 melanoma cells. (A) Laser scanning confocal microscopy (LSCM) revealed that F-actin appeared organized in numerous clear stress fibers crossing the cytoplasm in non-irradiated cells. Following narrow-band UVB irradiation (100 mJ/cm^2^), these stress fibers became obscure as F-actin was disassembled. This event could be observed as early as (B) 30 min, becoming more evident at (C) 60 min, with the appearance of punctate spots at (D) 6 h.

The changes in the cytoskeletal F-actin were observed using an LSCM. F-actin appeared to be organized in numerous clear stress fibers crossing the cytoplasm in non-irradiated cells (Fig. 2A). However, these stress fibers became obscure as actin was disassembled following narrow-band UVB irradiation (100 mJ/cm^2^) in a time-dependent manner. This event was observed as early as 30 min (Fig. 2B) after irradiation and became more evident with the appearance of punctate spots at 6 h (Fig. 2C and D).

### Effects of narrow-band UVB radiation on the activity of GTP-Rac1 and -RhoA in B16 melanoma cells

**Figure 3:**
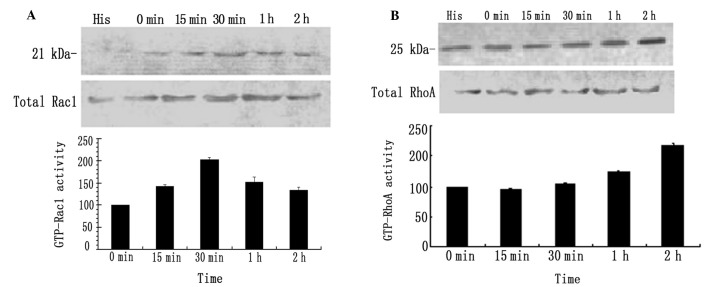
Effects of narrow-band UVB radiation on the activity of GTP-RhoA and -Rac1 in B16 melanoma cells. (A) The pull-down analysis revealed that the GTP-Rac1 protein expression was significantly increased at 15 min and by 2-fold at 30 min following narrow-band UVB irradiation (100 mJ/cm^2^), followed by a minor decrease, although the protein levels remained elevated at 60 and 120 min compared to control cells. (B) GTP-RhoA levels were marginally changed during the first 30 min after irradiation, were elevated at 60 min and by 1.6-fold at 120 min.

The pull-down analysis (Fig. 3A) revealed that GTP-Rac1 protein expression was significantly increased at 15 min and reached twice the baseline levels at 30 min after narrow-band UVB irradiation (100 mJ/cm^2^), followed by a minor decrease, although the protein levels remained elevated at 60 and 120 min compared to the control cells. The GTP-RhoA levels were marginally altered within the first 30 min after narrow-band UVB irradiation (100 mJ/cm^2^), were elevated at 60 min and by 1.6-fold at 120 min compared to the control cells (Fig. 3B).

## Discussion

Over the last few years, the molecular mechanisms responsible for melanocyte dendricity have attracted increasing attention in the field of melanocyte research. By examining the cytoskeletal components during cAMP-induced dentricity in B16F10 cells, it was observed that the cAMP-dependent dendricity was accompanied by a significant reorganization of the actin cytoskeleton (21). It has been verified previously that UV light irradiation is involved in the regulation of dendricity in human or mice melanocytes, as described above (3,18–20).

In the present study, the effect of narrow-band UVB radiation on morphological changes in B16 melanoma cells *in vitro* was observed. Marked morphological changes were evident in B16 melanoma cells treated with 100 mJ/cm^2^ narrow-band UVB radiation, with globular cell bodies and increased numbers of tree branch-like dendrites as compared to untreated cells, which merely exhibited 2–3 dendrites. Furthermore, the changes in the cytoskeletal F-actin microfilament in response to narrow-band UVB radiation were investigated using LSCM. LSCM revealed that F-actin appeared organized in numerous clear stress fibers crossing the cytoplasm in non-irradiated cells. However, these stress fibers became obscure as actin was disassembled following narrow-band UVB irradiation in a time-dependent manner. This event was observed as early as 30 min following irradiation and became more evident with the appearance of punctate spots at 6 h. These results suggest that narrow-band UVB radiation may promote the disorganization of cytoskeletal F-actin, leading to morphological changes such as globular cell bodies and increased numbers of dendrites in B16 melanoma cells.

The small GTPases, including RhoA, Rac1 and Cdc42, have been shown to regulate the assembly and disassembly of the actin cytoskeleton in neural crest-derived cells (3,21). Another possible explanation for the morphological changes exhibited by B16 melanoma cells exposed to narrow-band UVB radiation is the up- or downregulation of these small GTPases, which is based on the findings of previous studies, according to which Rac1 activation and RhoA inhibition induce elongation of the dendrites and the activated RhoA stimulates dendrite retraction (5,22–24).

In this study, we investigated the effect of narrow-band UVB radiation on Rac1 and RhoA activity using the pull-down assay. The analysis revealed that GTP-Rac1 protein expression was significantly increased at 15 min and had doubled at 30 min following narrow-band UVB irradiation, which was followed by a minor decrease, although the protein levels remained elevated at 1 and 2 h compared to non-irradiated cells. GTP-RhoA expression exhibited minor alterations in the first 30 min following narrow-band UVB irradiation, was elevated at 1 h and by 1.6-fold at 2 h compared to control cells. Unlike the overexpression of Rac1, RhoA activity in B16 melanoma cells exhibited no significant change in the short period after narrow-band UVB irradiation, although a delayed elevation was observed. The reason for this finding has not been elucidated. Ridley *et al*(25) concluded that a linear hierarchy exists, with Rac activating Rho, which appears to be consistent with the results of our study. It was hypothesized that Rac-dependent Rho activation in response to narrow-band UVB irradiation may serve as a negative feedback to avoid exorbitant dendrite extension promoted by the activated Rac1, according to the role of RhoA activation in dendrite retraction in B16 melanoma cells (23). In summary, our results suggest that narrow-band UVB radiation specifically activates the Rac1 signaling pathway, leading to F-actin rearrangement and resulting in increased dendrite formation in B16 melanoma cells.
